# Patient and general population values for luminal and perianal fistulising Crohn’s disease health states

**DOI:** 10.1007/s10198-019-01065-y

**Published:** 2019-05-17

**Authors:** Fanni Rencz, Peep F. M. Stalmeier, Márta Péntek, Valentin Brodszky, Gábor Ruzsa, Lóránt Gönczi, Károly Palatka, László Herszényi, Eszter Schäfer, János Banai, Mariann Rutka, László Gulácsi, Peter L. Lakatos

**Affiliations:** 1 Department of Health Economics, Corvinus University of Budapest, Fővám tér 8, 1093 Budapest, Hungary; 2 Premium Postdoctoral Research Program, Hungarian Academy of Sciences, Nádor u. 7, 1051 Budapest, Hungary; 3 Radboud University Medical Centre, PO Box 9101, 6500 HB Nijmegen, The Netherlands; 4 Doctoral School of Psychology, Institute of Psychology, Eötvös Loránd University of Sciences, Izabella u. 46, 1064 Budapest, Hungary; 5 Department of Statistics, Corvinus University of Budapest, Fővám tér 8, 1093 Budapest, Hungary; 6 1st Department of Medicine, Semmelweis University, Korányi Sándor u. 2/a, 1083 Budapest, Hungary; 7 Division of Gastroenterology, Department of Internal Medicine, University of Debrecen, Nagyerdei krt. 98, 4032 Debrecen, Hungary; 8 Medical Centre, Hungarian Defence Forces, Podmaniczky u. 109-111, 1062 Budapest, Hungary; 9 1st Department of Internal Medicine, University of Szeged, Korányi fasor 8-10, 6720 Szeged, Hungary; 10 Division of Gastroenterology, McGill University, MUHC, Montreal General Hospital, 1650 Ave. Cedar, D16.173.1, Montreal, QC H3G 1A4 Canada

**Keywords:** Crohn’s disease, Perianal fistula, Quality of life, Time trade-off, Utility, QALY, Hungary, I10

## Abstract

**Background:**

In patients with Crohn’s disease (CD), luminal disease activity paralleled by perianal fistulas may seriously impair health-related quality of life (HRQoL). Health utility values are not available from patients with CD that reflect the health loss associated with both luminal and perianal CD.

**Objective:**

To generate utilities for luminal and concomitant perianal fistulising CD health states directly from patients and from members of the general public.

**Methods:**

A cross-sectional survey was undertaken enrolling CD patients and a convenience sample of members of the general population. Respondents were asked to evaluate four common CD heath states [severe luminal disease (sCD), mild luminal disease (mCD), severe luminal disease with active perianal fistulas (sPFCD), and mild luminal disease with active perianal fistulas (mPFCD)] by 10-year time trade-off (TTO). In addition, patients assessed their current HRQoL by the TTO method.

**Results:**

Responses of 206 patients (40.8% with perianal fistulas) and 221 members of the general population were analysed. Mean ± SD utilities among patients for sPFCD, sCD, mPFCD and mCD states were 0.69 ± 0.33, 0.73 ± 0.31, 0.80 ± 0.29 and 0.87 ± 0.26. Corresponding values in the general public were: 0.59 ± 0.31, 0.65 ± 0.29, 0.80 ± 0.26 and 0.88 ± 0.25. Patients with active perianal fistulas, previous non-resection surgeries, and higher pain intensity scores valued their current health as worse (*p* < 0.05).

**Conclusions:**

TTO is a feasible method to assess HRQoL in patients with perianal fistulising disease, often not captured by health status questionnaires. Utilities from this study are intended to support the optimization of treatment-related decision making in patients with luminal disease paralleled by active perianal fistulas.

**Electronic supplementary material:**

The online version of this article (10.1007/s10198-019-01065-y) contains supplementary material, which is available to authorized users.

## Introduction

Crohn’s disease (CD) is a chronic inflammatory disorder of the gastrointestinal tract, often characterised by potentially debilitating symptoms such as abdominal pain, rectal bleeding, diarrhoea, fatigue and urgency [1]. Perianal fistulising Crohn’s disease (PFCD) is a common manifestation of CD affecting up to 40% of all patients [2]. In the majority of cases, perianal disease is paralleled by luminal disease activity. Symptoms of PFCD include pain, scarring, discharge, faecal incontinence and sexual difficulties. In addition to physical symptoms associated with CD, there may be adverse psychosocial effects [3, 4]. The resulting morbidity may seriously compromise health-related quality of life (HRQoL) and employment status of patients [5–7]. Despite the large burden of disease, HRQoL in PFCD has been less documented compared to luminal CD [8–10].

Few HRQoL studies have elicited health utilities for CD health states using a direct method [11]. Utilities are values measured on a cardinal scale anchored on 0 (= death) and 1 (= full health), which measure the impact of an illness on HRQoL [12, 13]. These utilities are required in cost-effectiveness analyses to calculate health benefits of a treatment expressed as quality-adjusted life years (QALYs). In the past 2 decades, biological drugs revolutionised the treatment of both luminal and fistulising CD [14, 15]; nevertheless, no reliable directly elicited health utilities are present for the cost-effectiveness analyses of biological drugs [16, 17].

Utility studies in CD were mostly conducted about 2 decades ago before or in the early era of biological drugs [18–20], often had a low sample size (< 50 patients) [18, 20, 21], concerned only luminal disease but not PFCD [19, 20], or suffered from a number of methodological shortcomings [21]. A very recent study from the UK used both patients and a general population sample to derive utilities for various PFCD health states [22]. In this study, luminal disease severity was constant for all health states, and the study focused on surgical outcomes of perianal fistulas. The utilities elicited may be less useful for cost-effectiveness models of biological drugs that are indicated to be effective for treating both luminal and PFCD. Therefore, utilities reflecting the health loss associated with luminal and concomitant perianal disease are needed.

The present study was designed to (1) generate utilities for common CD health states featuring luminal or luminal and concomitant perianal disease from the perspective of patients and members of the general public; (2) compare utilities for luminal CD to those when parallel perianal symptoms are present; (3) explore the impact of perspectives on health utilities, and (4) determine whether utilities are related to respondents’ demographics, health status or clinical characteristics.

## Methods

### Study design

Two cross-sectional surveys were undertaken. First, a paper-based survey was conducted with patients diagnosed with CD regardless of having perianal fistulising disease. The second, Internet-based, survey included members of the general population. Permission for conducting the study was granted by the National Scientific and Ethical Committee (Reference no. 49548-4/2016/EKU). An informed consent form was signed by all participants.

#### Data collection: patient survey

Data were collected between October 2016 and September 2017. Consecutive outpatients over 18 years from three academic gastroenterology departments and an inflammatory bowel diseases (IBD) centre in Hungary were enrolled in the study. The survey comprised of a paper-based questionnaire, the first part of which was completed by the patients, and the second by their gastroenterologist. Patients were asked about their socio-demographic characteristics and health status. CD-related pain intensity was recorded on a horizontal visual analogue scale (VAS) with the endpoints of ‘no pain at all’ (= 0) and ‘pain as bad as it could be’ (= 10).

In the second part of the survey, gastroenterologists provided data about the medical history, clinical characteristics and treatments of their patients. Gastroenterologists assessed disease severity using Crohn’s Disease Activity Index (CDAI) and Perianal Disease Activity Index (PDAI) [23, 24]. The CDAI is primarily based on a list of clinical symptoms or laboratory findings in the past 7 days. CDAI total scores range from 0 to 600, where a higher score represents a more severe disease. The PDAI includes five items (i.e., discharge, pain/restriction of activities, restriction of sexual activity, type of perianal disease and degree of induration). Total PDAI score varies between 0 and 20, where a score of ≤ 4 identifies an inactive disease, whereas a PDAI of > 4 suggests an active fistulising disease [24, 25].

#### Data collection: general population survey

Internet-based questionnaires were completed between November 2017 and March 2018. A convenience sample of the general population (aged ≥ 18 years) was recruited from the campus of Corvinus University of Budapest to participate in the survey. Participation was voluntary, anonymous and no remuneration was offered. The questionnaire collected data on socio-demographic characteristics of the respondents as well as their prior knowledge about CD (e.g., have known someone with CD). All questions of the online survey were mandatory, so respondents could not proceed to the next question without answering the previous one.

### Utility assessment

#### Health state vignettes

Four health state vignettes were designed: severe luminal disease (sCD), mild luminal disease (mCD), severe luminal disease with active perianal fistulas (sPFCD), and mild luminal disease with active perianal fistulas (mPFCD). The descriptions were developed by a group of IBD experts and health economists experienced in utility assessment. The final vignettes combined a description of living with CD including intestinal symptoms, abdominal pain, fistula symptoms, sleep, extraintestinal symptoms, eating, work/school, and leisure and social activities (Supplementary material S1). The descriptions were presented from a first-person perspective in a table format, as an earlier study reported that patients strongly prefer this format over narrative health state descriptions [26]. Subjects were asked to read the descriptions carefully and imagine living in the condition described.

#### Time trade-off (TTO)

All TTO tasks were self-administered. The TTO method elicits HRQoL or utility values for imperfect health states by asking respondents to make a trade off between quality and length of life [13]. We opted to use a 10-year time frame, as this is most commonly used for the valuation of health states [27–32]. Individuals were asked to imagine living in the CD health states as described in the vignettes for the ensuing 10 years, followed by death. Then they had to indicate how many life years they would give up to regain full health. Respondents were offered to choose from 20 predefined, tradable amounts of time (0 years, 6 months, 1 year,…, 9 years, 9.5 years and 10 years). An example for a TTO valuation task is provided in Supplementary material S2. The four health states were randomized within respondents both for patients and the general population sample.

TTO utilities were calculated according to the following formula:

$$ {\text{Utility}} = 1 - {\text{disutility}} = 1 - \frac{ {\text{participant}}^{\prime}{\text{s}}\;{\text{answer}} }{ 10 \;{\text{years}} }. $$

Suppose, for example, a respondent indicated to exchange 2 years, yielding *U* = (10 − 2)/10 = 0.8. Therefore, TTO utilities in this study were anchored on 0 (death) and 1 (full health).

### Statistical analyses

Socio-demographic and clinical characteristics of the patient and general population groups were compared using a Student’s *t* test and *χ*^2^ test. All non-missing TTO responses were included in the primary data analysis. Descriptive statistics (mean, median, standard deviation and IQR) of utilities were computed. A paired *t* test was used to test the difference between TTO utilities for hypothetical health states. TTO utilities within subgroups of patients were compared by Student’s *t* test or analysis of variance (ANOVA), where applicable.

We performed random-effects linear regression models to explore demographic, clinical and other possible predictors of utilities for hypothetical health states. Before running the models, the following participants were excluded: (1) non-traders (i.e., who valued all health states equal to full health), (2) who indicated the same value for all health states, (3) who had more than two missing TTO responses out of the four hypothetical health states and (4) who indicated a logical inconsistency (i.e., utilities for sCD > mCD or sPFCD > mPFCD or sPFCD > mCD). Determinants of utilities for current health were analysed by ordinary least squares regression. No exclusions were applied before the regression analysis of utilities for current health. All the statistics were two-sided, and *p* < 0.05 was considered statistically significant. Data were analysed using Stata 13 (College Station, TX, USA: StataCorp LP).

## Results

### Demographic and clinical characteristics of the study populations

Overall, 206 patients with CD and 221 adults from the general population participated in the study. There were a total of 41 (4.0%) missing TTO responses from patients: *n* = 7 for sCD, *n* = 7 for mCD, *n* = 9 for sPFCD, *n* = 13 for mPFCD and *n* = 5 for current health. There were no missing responses in the general population sample.

Table 1 provides demographic characteristics of the study participants. Patients and members of the general population were similar with respect to demographics. Patients were on average 3 years younger compared to the general population. There was a slight male predominance in both groups. A total of 28.8% of patients had a tertiary education, while this rate was 56.1% for the general population sample. The distribution of participants in the two groups was more or less balanced according to employment status and place of residence within the country.

**Table 1:** Demographics and general health in the study populations

Variables	Mean (SD) or *N* (%)		*p* value
	Patients with CD (*n* = 206)	General population sample (*n* = 221)	
Sex			
Female	93 (45.1%)	71 (32.1%)	< 0.001
Male	113 (54.9%)	150 (67.9%)	
Age (years)	34.7 (10.5)	37.3 (15.5)	< 0.001
Age groups (years)			
18–24	37 (18.0%)	75 (33.9%)	< 0.001
25–34	71 (34.5%)	39 (17.6%)	
35–44	59 (28.6%)	27 (12.2%)	
≥ 45	39 (18.9%)	80 (36.2%)	
Education (*n* = 1)			
Primary school	14 (6.8%)	4 (1.8%)	< 0.001
Secondary school	132 (71.2%)	93 (42.1%)	
College/university	59 (28.8%)	124 (56.1%)	
Employment			
Student	25 (12.1%)	63 (28.5%)	< 0.001
Full time	110 (53.4%)	100 (45.2%)	0.092
Part time	30 (14.6%)	21 (9.5%)	0.107
Unemployed	11 (5.3%)	9 (4.1%)	0.536
Retired	3 (1.5%)	12 (5.4%)	0.026
Disability pensioner	49 (23.8%)	4 (1.8%)	< 0.001
Other	15 (7.3%)	12 (5.4%)	0.424
Place of residence			
Capital (Budapest)	45 (21.8%)	102 (46.2%)	< 0.001
County town	34 (16.5%)	24 (10.9%)	0.089
Smaller town	81 (39.3%)	63 (28.5%)	0.018
Village	46 (22.3%)	32 (14.5%)	0.036
Subjective life expectancy (years)	76.3 (12.4)	79.0 (11.0)	0.019
Prior experiences with CD of participants from the general population			
Have never heard about it	N/A	153 (69.2%)	N/A
Have heard about it^a^	N/A	68 (30.8%)	N/A
Have been diagnosed with CD	N/A	8 (3.6%)	N/A
Have a family member or acquaintance with CD	N/A	9 (4.1%)	N/A
Doctor	N/A	9 (4.1%)	N/A
Employed in healthcare (but not a doctor)	N/A	10 (4.5%)	N/A
Medical student	N/A	1 (0.5%)	N/A
Read/heard about it from the Internet/media/newspaper	N/A	37 (16.7%)	N/A
Other	N/A	5 (2.3%)	N/A

^a^One person may have heard about it from multiple sources

*CD* Crohn’s disease, *N/A* not applicable

More than two-thirds of the general population sample have never heard about CD. Altogether, 16.7% have read or heard about CD from the internet/media/newspapers, 8.6% learned about CD as they were being employed in healthcare, 4.1% had a family member or acquaintance suffering from CD and 3.6% indicated to be diagnosed with CD (Table 1).

Table 2 presents the clinical characteristics of the patient sample. Mean ± SD disease duration was 10.5 ± 6.3 years. Eighty-four patients (41%) had perianal fistulas, 36.1% of them being active. Extraintestinal manifestations were present in 57 patients (27.7%). At the time of the survey, 66% received biological therapy (infliximab 47.6%, adalimumab 17.5% and vedolizumab 1.9%).

**Table 2:** Clinical characteristics of CD patients (*n* = 206)

	Mean (SD) or *N* (%)
Body mass index, BMI (kg/m^2^) (missing *n* = 2)	23.4 (4.3)
Underweight (BMI > 18.5)	22 (10.8%)
Normal (BMI 18.5–24.9)	111 (54.4%)
Overweight (BMI 25.0–29.9)	57 (27.9%)
Obesity (BMI > 30)	14 (6.9%)
Extraintestinal manifestations	57 (27.7%)
Disease severity: CDAI (0–600)	110.5 (77.0)
Symptomatic remission (CDAI < 150)	156 (75.7%)
Mild (CDAI 150–219)	32 (15.5%)
Moderate to severe (CDAI 220 ≤)	18 (8.7%)
Perianal fistula severity: PDAI (0–20) (missing *n* = 1)	3.68 (2.29)
Inactive (PDAI ≤ 4)	53 (63.9%)
Active (PDAI > 4)	30 (36.1%)
Pain	
Pain VAS (0–100)	24.7 (23.9)
Current treatment	
None	3 (1.5%)
Systemic non-biological	67 (32.5%)
Biological	136 (66.0%)
Previous surgeries due to CD^a^	
None	87 (42.2%)
Resection surgery	66 (32.0%)
Non-resection surgery^b^	86 (41.7%)

*BMI* body mass index, *CD* Crohn’s disease, *CDAI* Crohn’s Disease Activity Index, *PDAI* Perianal Disease Activity Index, *VAS* visual analogue scale

^a^Overall, 33 patients had both resection and non-resection surgeries

^b^Perianal fistula surgery, strictureplasty or abscess drainage

### Utility results

Table 3 shows the mean utilities derived from the two groups for the hypothetical health states as well as current health for patients. Among patients, mean utilities were 0.69 ± 0.33 for sPFCD, 0.73 ± 0.31 for sCD, 0.80 ± 0.29 for mPFCD and 0.87 ± 0.26 for mCD health state. Corresponding values in the general public were as follows: 0.59 ± 0.31, 0.65 ± 0.29, 0.80 ± 0.26 and 0.88 ± 0.25. In both groups, significant differences were observed across all hypothetical health states (*p* < 0.001).

**Table 3:** TTO values for current health and hypothetical CD health states

Health state	Patients					General population					*p* value^*^
	*N*	Mean (SD)	Median (IQR)	‘0’ utilities (*N*, %)	‘1’ utilities (*N*, %)	*N*	Mean (SD)	Median (IQR)	‘0’ utilities (*N*, %)	‘1’ utilities (*N*, %)	
Severe luminal disease (sCD)	199	0.73 (0.31)	0.90 (0.50–1)	4 (2.0%)	60 (30.2%)	221	0.65 (0.29)	0.70 (0.50–0.90)	13 (5.9%)	22 (10.0%)	0.005
Mild luminal disease (mCD)	199	0.87 (0.26)	1 (0.90–1)	3 (1.5%)	116 (58.3%)	221	0.88 (0.25)	1 (0.90–1)	10 (4.5%)	123 (55.7%)	0.919
Severe luminal disease with active perianal fistulas (sPFCD)	197	0.69 (0.33)	0.80 (0.50–1)	8 (4.1%)	55 (27.9%)	221	0.59 (0.31)	0.65 (0.40–0.85)	17 (7.7%)	20 (9.0%)	0.002
Mild luminal disease with active perianal fistulas (mPFCD)	193	0.80 (0.29)	0.90 (0.70–1)	5 (2.6%)	74 (38.3%)	221	0.80 (0.26)	0.90 (0.80–1)	8 (3.6%)	66 (29.9%)	0.794
Current health	201	0.83 (0.28)	1 (0.80–1)	4 (2.0%)	103 (51.2%)	N/A	N/A	N/A	N/A	N/A	N/A

*CD* Crohn’s disease, *N/A* not applicable, *TTO* time trade-off

*p < 0.05 indicates statistically significant difference between the two groups

For each health state, the proportion of patients not willing to give up any time (i.e., ‘1’ answers) is presented in Table 3. Among patients, this rate ranged from 27.9% for sPFCD to 58.3% for mCD. In contrast, these figures were 9.0% and 55.9% for the general public. There were 50 patients (24.3%) and 26 members of the general population (11.8%) who were non-traders (i.e., rated all health states equal to ‘1’, including their current health for patients). Overall, 4.1%, 2.0%, 2.6% and 1.5% rated the sPFCD, sCD, mPFCD and mCD to be as bad as dead (utility = 0).

Mean TTO utilities from patients were higher for the two severe health states compared to the general public (*p* < 0.01). There was no statistically significant difference in TTO scores for the two mild health states between the two groups.

Table 4 demonstrates the mean current health state utility for subgroups of patients. Mean utility for patients’ current health was 0.83 ± 0.28. The TTO method well discriminated between patients with active perianal fistulas and those with inactive or no fistulas (0.67 ± 0.37 vs. 0.86 ± 0.26; *p* = 0.013). Previous non-resection (0.76 ± 0.33) or both resection and non-resection (0.75 ± 0.37) surgeries were associated with lower mean TTO values (*p* = 0.023). Differences between groups based on CDAI score also showed a trend towards statistical significance.

**Table 4:** TTO utilities for current health in subgroups of patients (*n* = 201)

Variables	Groups	*N*	Mean (SD)	*p* value
Disease severity	Symptomatic remission (CDAI < 150)	153	0.85 (0.27)	0.086
	Mild (CDAI 150–219)	31	0.84 (0.33)	
	Moderate to severe (CDAI 220 ≤)	17	0.69 (0.34)	
Perianal fistulas	Inactive (PDAI ≤ 4) or no	172	0.86 (0.26)	0.013
	Active (PDAI > 4)	29	0.67 (0.37)	
Extraintestinal manifestations	No	146	0.85 (0.26)	0.858
	Yes	55	0.82 (0.29)	
Current treatment	Non-biological	66	0.87 (0.25)	0.139
	Biological	132	0.81 (0.30)	
Previous surgeries due to CD	None	86	0.89 (0.20)	0.023
	Resection	31	0.87 (0.26)	
	Non-resection	51	0.76 (0.33)	
	Both	33	0.75 (0.37)	

*CD* Crohn’s disease, *CDAI* Crohn’s Disease Activity Index, *PDAI* Perianal Disease Activity Index, *TTO* time trade-off

### Multivariate analysis of predictors of utilities

Supplementary material S3 demonstrates the results of regression models about predictors of TTO utilities. Patients’ utilities for the hypothetical health states were decreased by 0.012 with 1-point increase in body mass index (BMI) (*p* = 0.005). One-point increase in disease severity on CDAI resulted in a 0.001 decrease in utilities (*p* = 0.036). Employment status of patients had a significant impact on utilities, those working full time indicated lower values by 0.071 (*p* = 0.020).

With respect to patients’ current health, each 1-point increase in pain intensity on a VAS (scale range 0–10) was associated with a 0.032 decrease in TTO utilities (*p* < 0.001). Patients who previously had a non-resection surgery due to CD rated hypothetical health states to 0.095 lower (*p* = 0.015). Patients who were retired valued their current health utility lower by 0.176, while those who were students gave higher values by, on average, 0.106.

Among members of the general public, TTO values for hypothetical health states increased by 0.002 with every 1-year increase in age and by 0.003 with every 1-year increase in subjective life expectancy (*p* = 0.018). People who had a family member or acquaintance diagnosed with CD valued health states higher (+ 0.111, *p* = 0.043), while disability pensioners rated them to be lower by 0.174 (*p* = 0.021).

## Discussion

### Main findings

This study evaluated individual preferences for different health states of CD using patients and a convenience sample from the Hungarian general population. The mean TTO values ranged between 0.69 and 0.88 derived from patients, and between 0.59 and 0.88 from the general population. In both groups, utilities for health states of severe and mild fistulising disease were significantly lower compared with health states describing the same luminal disease severity but with no concomitant perianal fistula symptoms. Patients assigned significantly higher values to the two health states of mild luminal severity than the general population, but this was not true for the severe health states. Patients with active perianal fistulas valued their current health much lower compared to those with inactive or no fistulas. Higher pain intensity experienced along with previous non-resection surgeries including perianal fistula surgery, strictureplasty and abscess drainage, were important predictors of reporting lower utilities for current health.

### Comparison with other studies

Our results concur with findings from previous studies indicating that perianal fistulas have a considerable impact on HRQoL [5]. Similarly to our study, Longworth et al. [22] used a 10-year time frame to assess TTO utilities for perianal fistulising health states in the UK. In their study, patients and the general public valued sPFCD health state to a mean of 0.38 and 0.43, while mPFCD resulted in a mean of 0.58 and 0.66, respectively. In comparison, we found higher mean utilities for both sPFCD (patients: 0.69 and general public: 0.59) and mPFCD (both patients and general population: 0.80). The differences may be attributable to the different patient populations in terms of disease characteristics and severity as well as to the methodological variations of TTO between the two studies. For instance, the UK study [22] employed different health state vignettes and allowed to value health states worse than dead.

### Strengths and limitations

This is the largest study involving patients to directly elicit utilities for CD health states. Furthermore, compared to previous studies, health state vignettes used in the present study were more comprehensive featuring all aspects of HRQoL that may be relevant to CD patients. Another strength is the heterogeneous patient population recruited with regard to demographic as well as clinical characteristics, which satisfies the purpose of this valuation study. The study has limitations as well. First, two-thirds of the patients were treated with biological drugs and, as a result, they were in a relatively good health state. Second, there were differences in socio-demographic characteristics such as age and educational background between patients and the general population sample. Furthermore, our sample was on average younger, and had a higher proportion of male and highly educated participants compared to a nationally representative sample of the Hungarian general population (mean age 42 years, males 47%, college/university graduates 21%) [33].

### Practical implications

The TTO seems to be superior to indirect utility assessment when it comes to PFCD. As shown by earlier studies, the EQ-5D-3L or EQ-5D-5L questionnaires could not distinguish between subgroups of CD patients based on perianal disease [6, 34]. We found that the 10-year TTO method discriminated well between HRQoL of patients with active and inactive or no perianal fistulas. In cost-effectiveness analyses of treatments for PFCD, directly elicited utilities can be recommended to be used to calculate QALYs. Before this study, in absence of relevant and reliable utility data specifically for PFCD, all published cost-effectiveness analyses of biological drugs relied on either luminal CD results or unpublished data such as expert opinion [35–37]. Our results fill in the gap in literature by providing robust health utilities from both patient and general population perspectives for the economic evaluations of biological drugs for CD.

In many countries including the US, Canada, the UK, the Netherlands and Hungary a general population perspective is recommended in the context of economic evaluations, while other countries, such as Sweden, recommend the patient perspective [38–42]. There is an increasing body of literature arguing that utilities based on both patient and general public preferences should be considered in health technology assessment [43–46]. A result with direct impact on cost-effectiveness studies of our study is that the differences in utilities between the two severe and mild states were found to be smaller for patients compared to those among members of the general population. This implies that using utilities reflecting patient preferences in cost-effectiveness analyses may reduce the QALY gain associated with therapy.

The assessment of HRQoL is advocated in guidelines for the management of PFCD by professional societies such as the European Crohn’s and Colitis Organisation (ECCO) and the American College of Gastroenterology (ACG) [47–49]. However, currently there is no validated, disease-specific, patient-derived HRQoL tool for PFCD. Development of such an outcome measure, Crohn’s Anal Fistula Quality of Life (CAF-QoL) is underway [50], although, the final version was not available at the time this manuscript was written. Importantly, we found that pain intensity has a large impact on HRQoL in patients with CD. Current ECCO and ACG guidelines recommend measuring abdominal pain as a part of the CDAI score. Our findings point out that thorough pain assessment would be essential in improving patients’ HRQoL.

## Conclusions

Overall, the results of this study well reflect the severity of different health states of CD, and highlight the additional HRQoL burden of living with perianal fistulising disease. It seems that the TTO method offers an accurate assessment of HRQoL in patients with PFCD, often not captured by health status questionnaires. Utilities from the present study are intended to support the optimization of treatment-related decision making in patients with luminal disease paralleled by active perianal fistulas and to establish a solid basis for cost-effectiveness analyses to compare treatment strategies.

## Electronic supplementary material

Below is the link to the electronic supplementary material.

Supplementary material 1 (PDF 217 kb)

Supplementary material 2 (PDF 231 kb)

Supplementary material 3 (PDF 234 kb)
