# Supplementary material for: Patient and general population values for luminal and perianal fistulising Crohn’s disease health states
Source: Eur J Health Econ. 2019 May 17;20(Suppl 1):91–100. doi: 10.1007/s10198-019-01065-y (PMC6544586; doi:10.1007/s10198-019-01065-y)
Supplement: Supplementary file 1 — Supplementary material 1 (PDF 217 kb) [file 10198_2019_1065_MOESM1_ESM.pdf]

Supplementary material S1 Health state vignettes

Journal: European Journal of Health Economics (2019)

Title: Patient and general population values for luminal and perianal fistulising Crohn's disease health states

Authors: Fanni Rencz[1,2], Peep F.M. Stalmeier[3], Márta Péntek[1], Valentin Brodszky[1], Gábor Ruzsa[4,5], Lóránt Gönczi[6], Károly Palatka[7], László Herszényi[8], Eszter Schäfer[8], János Banai[8], Mariann Rutka[9], László Gulácsi[1]* and Peter L. Lakatos[6,10]*

1– Department of Health Economics, Corvinus University of Budapest, Fővám tér 8, H-1093, Budapest, Hungary

2 – Hungarian Academy of Sciences, Premium Postdoctoral Research Program, Nádor u. 7, H-1051 Budapest, Hungary

3 – Radboud University Medical Centre, PO Box 9101, 6500 HB, Nijmegen, The Netherlands

4 – Eötvös Loránd University of Sciences, Institute of Psychology, Doctoral School of Psychology, Izabella u. 46, H-1064, Budapest, Hungary

5 – Department of Statistics, Corvinus University of Budapest, Fővám tér 8, H-1093, Budapest, Hungary

6 – 1st Department of Medicine, Semmelweis University, Korányi Sándor u. 2/a, H-1083 Budapest, Hungary

7 – Division of Gastroenterology, Department of Internal Medicine, University of Debrecen, Nagyerdei krt. 98, H-4032 Debrecen, Hungary

8 – Medical Centre, Hungarian Defence Forces, Podmaniczky u. 109-111, H-1062 Budapest, Hungary

9 – 1st Department of Internal Medicine, University of Szeged, Korányi fasor 8-10, H-6720 Szeged, Hungary

10 – Division of Gastroenterology, McGill University, MUHC, Montreal General Hospital, 1650 Ave. Cedar, D16.173.1, Montreal, QC, H3G 1A4, Canada

Corresponding author: Fanni Rencz, fanni.rencz@uni-corvinus.hu

Supplementary material S1 Health state vignettes

	Severe luminal disease	Mild luminal disease	Severe luminal disease with active perianal fistulas	Mild luminal disease with active perianal fistulas
Intestinal symptoms, abdominal pain	You have frequent diarrhoea or loose stools, from 5-8 per day. You often have urgent stools and sometimes experience episodic faecal incontinence. You regularly take anti-diarrheal medications.<br>When you have diarrhoea/loose stools, you often get moderate to severe abdominal cramping. Abdominal pain episodes may occur a few times a day (either independently from stools) and they may take for a while (more than 30 minutes), so you are occasionally or frequently on painkillers.	You have loose stools, a maximum of 2-4 per day. You are able to control stools and rarely have urgent stools.<br>When you have loose stools, you rarely get mild abdominal cramping (less than 5 minutes). You do not need painkillers.	You have frequent diarrhoea or loose stools, from 5-8 per day. You often have urgent stools and sometimes experience episodic faecal incontinence. You regularly take anti-diarrheal medications.<br>When you have diarrhoea≥/loose stools, you often get moderate to severe abdominal cramping. Abdominal pain episodes may occur a few times a day (either independently from stools) and they may take for a while (more than 30 minutes), so you are occasionally or frequently on painkillers.	You have loose stools, a maximum of 2-4 per day. You are able to control stools and rarely have urgent stools.<br>When you have loose stools, you rarely get mild abdominal cramping (less than 5 minutes). You do not need painkillers.
Fistula symptoms	You have no fistulas.*	You have no fistulas.*	You have perianal fistulas. These are small openings in the skin surface of the perianal region that allow continuous drainage, so you use pads inside underwear that you need to change a few times a day. The fistulas are inflamed and painful regardless of stools.	You have perianal fistulas. These are small openings in the skin surface of the perianal region that allow continuous drainage, so you use pads inside underwear that you need to change a few times a day. The fistulas are inflamed and painful regardless of stools.
Sleep	Your bowel symptoms usually cause you sleepless and restless nights. You are often feeling fatigued.	You experience no or very mild bowel symptoms at night, so you have no problems with sleeping.	Your bowel symptoms usually cause you sleepless and restless nights. You are often feeling fatigued.	You experience no or very mild bowel symptoms at night, so you have no problems with sleeping.
Extraintestinal symptoms	Extraintestinal symptoms may be present. Your joints are swollen and painful. Rarely	You have no joint, skin or eye symptoms.	Extraintestinal symptoms may be present. Your joints are swollen and painful. Rarely	You have no joint, skin or eye symptoms.

	painful inflammation of the skin (purulent or blistering) or the eyes may occur.		painful inflammation of the skin (purulent or blistering) or the eyes may occur.	
Eating	You often experience a lack of appetite. You are afraid of eating, because you are likely to experience urgency and abdominal pain following a meal. When you are leaving your home, you often avoid food to prevent symptoms. You may experience a considerable weight loss (5-15 kg / 2-6 months).	Food intake does not compromise your daily activities and rarely causes you diarrhoea or mild abdominal pain. You keep your weight up.	You often experience a lack of appetite. You are afraid of eating, because you are likely to experience urgency and abdominal pain following a meal. When you are leaving your home, you often avoid food to prevent symptoms. You may experience a considerable weight loss (5-15 kg / 2-6 months).	Food intake does not compromise your daily activities and rarely causes you diarrhoea or mild abdominal pain. You keep your weight up.
Work/school	Your symptoms cause you a considerable problem with accomplishing your work/school tasks, you usually have to miss days out of work/school due to your disease or physician visits/treatments.	You do not experience a problem with accomplishing your work/school tasks, you do not feel yourself fatigued. You rarely have a planned absence from work/school due to your disease or physician visits/treatments.	Your symptoms cause you a considerable problem with accomplishing your work/school tasks, you usually have to miss days out of work/school due to your disease or physician visits/treatments.	You do not experience a problem with accomplishing your work/school tasks, you do not feel yourself fatigued. You rarely have a planned absence from work/school due to your disease or physician visits/treatments.
Leisure and social activities	You need to cancel most of your leisure activities due to the symptoms. Your disease causes a limitation in your social relationships and sexual activity, you often feel embarrassed in front of others.	You do not have a problem with transportation or travelling. Your disease causes no limitation in your leisure and social activities. There is no need to cancel your leisure activities due to the symptoms. You rarely feel embarrassed in front of others.	You need to cancel most of your leisure activities due to the symptoms. Your disease causes a limitation in your social relationships and sexual activity, you often feel embarrassed in front of others.	You do not have a problem with transportation or travelling. Your disease causes no limitation in your leisure and social activities. There is no need to cancel your leisure activities due to the symptoms. You rarely feel embarrassed in front of others.

* fistula: small openings in the skin surface of the perianal region that may leak.
