# Supplementary material for: Patient and general population values for luminal and perianal fistulising Crohn’s disease health states
Source: Eur J Health Econ. 2019 May 17;20(Suppl 1):91–100. doi: 10.1007/s10198-019-01065-y (PMC6544586; doi:10.1007/s10198-019-01065-y)
Supplement: Supplementary file 2 — Supplementary material 2 (PDF 231 kb) [file 10198_2019_1065_MOESM2_ESM.pdf]

Supplementary material S2 Example for a TTO valuation task

Journal: European Journal of Health Economics (2019)

Title: Patient and general population values for luminal and perianal fistulising Crohn's disease health states

Authors: Fanni Rencz[1,2], Peep F.M. Stalmeier[3], Márta Péntek[1], Valentin Brodszky[1], Gábor Ruzsa[4,5], Lóránt Gönczi[6], Károly Palatka[7], László Herszényi[8], Eszter Schäfer[8], János Banai[8], Mariann Rutka[9], László Gulácsi[1]* and Peter L. Lakatos[6,10]*

1– Department of Health Economics, Corvinus University of Budapest, Fővám tér 8, H-1093, Budapest, Hungary

2 – Hungarian Academy of Sciences, Premium Postdoctoral Research Program, Nádor u. 7, H-1051 Budapest, Hungary

3 – Radboud University Medical Centre, PO Box 9101, 6500 HB, Nijmegen, The Netherlands

4 – Eötvös Loránd University of Sciences, Institute of Psychology, Doctoral School of Psychology, Izabella u. 46, H-1064, Budapest, Hungary

5 – Department of Statistics, Corvinus University of Budapest, Fővám tér 8, H-1093, Budapest, Hungary

6 – 1st Department of Medicine, Semmelweis University, Korányi Sándor u. 2/a, H-1083 Budapest, Hungary

7 – Division of Gastroenterology, Department of Internal Medicine, University of Debrecen, Nagyerdei krt. 98, H-4032 Debrecen, Hungary

8 – Medical Centre, Hungarian Defence Forces, Podmaniczky u. 109-111, H-1062 Budapest, Hungary

9 – 1st Department of Internal Medicine, University of Szeged, Korányi fasor 8-10, H-6720 Szeged, Hungary

10 – Division of Gastroenterology, McGill University, MUHC, Montreal General Hospital, 1650 Ave. Cedar, D16.173.1, Montreal, QC, H3G 1A4, Canada

Corresponding author: Fanni Rencz, fanni.rencz@uni-corvinus.hu

Supplementary material S2 Example for a TTO valuation task

Please carefully read through the following description about a health state.

Health state 'A'[*]

Intestinal symptoms, abdominal pain	You have frequent diarrhoea or loose stools, from 5-8 per day. You often have urgent stools and sometimes experience episodic faecal incontinence. You regularly take anti-diarrheal medications.<br>When you have diarrhoea/loose stools, you often get moderate to severe abdominal cramping. Abdominal pain episodes may occur a few times a day (either independently from stools) and they may take for a while (more than 30 minutes), so you are occasionally or frequently on painkillers.
Fistula symptoms	You have no fistulas.[§]
Sleep	Your bowel symptoms usually cause you sleepless and restless nights. You are often feeling fatigued.
Extraintestinal symptoms	Extraintestinal symptoms may be present. Your joints are swollen and painful. Rarely painful inflammation of the skin (purulent or blistering) or the eyes may occur.
Eating	You often experience a lack of appetite. You are afraid of eating, because you are likely to experience urgency and abdominal pain following a meal. When you are leaving your home, you often avoid food to prevent symptoms. You may experience a considerable weight loss (5-15 kg / 2-6 months).
Work/school	Your symptoms cause you a considerable problem with accomplishing your work/school tasks, you usually have to miss days out of work/school due to your disease or physician visits/treatments.
Leisure and social activities	You need to cancel most of your leisure activities due to the symptoms. Your disease causes a limitation in your social relationships and sexual activity, you often feel embarrassed in front of others.

[§] fistula: small openings in the skin surface of the perianal region that may leak.

Imagine that you live another 10 years health state 'A', and then you die. Suppose there is a treatment for this condition with which you may live for a smaller number of years, but in full health. What is the maximum number of years you would be willing to give up so that you can live in full health?

Please indicate your response with an X.

- ❑ 0 years (=none)
- ❑ 6 months
- ❑ 1 year
- ❑ 1 year 6 months
- ❑ 2 years
- ❑ 2 years 6 months
- ❑ 3 years
- ❑ 3 years 6 months
- ❑ 4 years
- ❑ 4 years 6 months
- ❑ 5 years
- ❑ 5 years 6 months
- ❑ 6 years
- ❑ 6 years 6 months
- ❑ 7 years
- ❑ 7 years 6 months
- ❑ 8 years
- ❑ 8 years 6 months
- ❑ 9 years
- ❑ 9 years 6 months
- ❑ 10 years (=immediate death)

[*] Note that health states were labelled as A, B, C and D in the questionnaire.
