# Supplementary material for: Patient and general population values for luminal and perianal fistulising Crohn’s disease health states
Source: Eur J Health Econ. 2019 May 17;20(Suppl 1):91–100. doi: 10.1007/s10198-019-01065-y (PMC6544586; doi:10.1007/s10198-019-01065-y)
Supplement: Supplementary file 3 — Supplementary material 3 (PDF 234 kb) [file 10198_2019_1065_MOESM3_ESM.pdf]

Supplementary material S3 Predictors of TTO utilities

Journal: European Journal of Health Economics (2019)

Title: Patient and general population values for luminal and perianal fistulising Crohn's disease health states

Authors: Fanni Rencz[1,2], Peep F.M. Stalmeier[3], Márta Péntek[1], Valentin Brodszky[1], Gábor Ruzsa[4,5], Lóránt Gönczi[6], Károly Palatka[7], László Herszényi[8], Eszter Schäfer[8], János Banai[8], Mariann Rutka[9], László Gulácsi[1]* and Peter L. Lakatos[6,10]*

1– Department of Health Economics, Corvinus University of Budapest, Fővám tér 8, H-1093, Budapest, Hungary

2 – Hungarian Academy of Sciences, Premium Postdoctoral Research Program, Nádor u. 7, H-1051 Budapest, Hungary

3 – Radboud University Medical Centre, PO Box 9101, 6500 HB, Nijmegen, The Netherlands

4 – Eötvös Loránd University of Sciences, Institute of Psychology, Doctoral School of Psychology, Izabella u. 46, H-1064, Budapest, Hungary

5 – Department of Statistics, Corvinus University of Budapest, Fővám tér 8, H-1093, Budapest, Hungary

6 – 1st Department of Medicine, Semmelweis University, Korányi Sándor u. 2/a, H-1083 Budapest, Hungary

7 – Division of Gastroenterology, Department of Internal Medicine, University of Debrecen, Nagyerdei krt. 98, H-4032 Debrecen, Hungary

8 – Medical Centre, Hungarian Defence Forces, Podmaniczky u. 109-111, H-1062 Budapest, Hungary

9 – 1st Department of Internal Medicine, University of Szeged, Korányi fasor 8-10, H-6720 Szeged, Hungary

10 – Division of Gastroenterology, McGill University, MUHC, Montreal General Hospital, 1650 Ave. Cedar, D16.173.1, Montreal, QC, H3G 1A4, Canada

Corresponding author: Fanni Rencz, fanni.rencz@uni-corvinus.hu

Supplementary material S3 Predictors of TTO utilities

Variables	Patient's current health TTO n=201, R^2=0.14			Patients' TTO for hypothetical health states[a] n=102, R^2=0.47			General population TTO for hypothetical heath states[b] n=155, R^2=0.55		
	Coefficient	Std. Error	p-value	Coefficient	Std. Error	p-value	Coefficient	Std. Error	p-value
Intercept	0.933	0.032	<0.001	0.749	0.103	<0.001	0.599	0.116	<0.001
Hypothetical health states							-	-	-
Severe luminal disease (sCD)	-	-	-	-0.215	0.017	<0.001	-0.299	0.016	<0.001
Mild luminal disease with active perianal fistulas (mPFCD)	-	-	-	-0.118	0.015	<0.001	-0.121	0.013	<0.001
Severe luminal disease with active perianal fistulas (sPFCD)	-	-	-	-0.312	0.019	<0.001	-0.393	0.016	<0.001
Demographics									
Age (years)	-	-	-	-	-	-	0.002	0.001	0.045
Employment									
Full-time	-	-	-	-0.071	0.031	0.020	-	-	-
Retired	-0.176	0.082	0.033	-	-	-	-	-	-
Student	0.106	0.053	0.047	-	-	-	-	-	-
Disability pensioner	-	-	-	-	-	-	-0.174	0.075	0.021
Clinical characteristics									
BMI	-	-	-	0.012	0.004	0.005	-	-	-
CDAI (0-600)	-	-	-	-0.001	0.0003	0.036	-	-	-
Pain VAS (0-10)	-0.032	0.008	<0.001	-	-	-	-	-	-
Previous non-resection surgery	-0.095	0.039	0.015	-	-	-	-	-	-
Other characteristics									
Family member or acquaintance diagnosed with CD	-	-	-	-	-	-	0.111	0.055	0.043
Subjective life expectancy (years)	-	-	-	-	-	-	0.003	0.001	0.018

a: Responses of 104 patients were excluded (n=50 non-traders, n=20 valued all health states for the same value, n=6 had more than two missing TTO responses and n=28 had illogical preferences). Thus, 102 patients were included in the regression analysis for hypothetical health states.
b: Responses of 66 people from the general public were excluded before regression analysis (n=26 non-traders and n=40 had illogical preferences). Thus, a total of 155 responses were analysed.

Independent variables: *Age* = respondent's age (years); *BMI* = body mass index (kg/m^2); *CDAI*: score on Crohn's Disease Activity Index (0-600); *Disability pensioner*: no=0, yes=1; *Family member or friend diagnosed with CD*: no=0, yes=1; *Full-time employed*: no=0, yes=1; *Pain VAS:* pain currently experienced on a 0-100 visual analogue scale; *Previous non-resection surgery*: no=0, yes=1; *Retired*: no=0, yes=1; *Student:* no=0, yes=1; *Subjective life expectancy* = the respondent's subjective life expectancy (years)
BMI = body mass index; CD = Crohn's disease; CDAI = Crohn's Disease Activity Index; TTO = time trade-off; VAS = visual analogue scale
